# 5-Azacytidine Inhibits the Activation of Senescence Program and Promotes Cytotoxic Autophagy during Trdmt1-Mediated Oxidative Stress Response in Insulinoma β-TC-6 Cells

**DOI:** 10.3390/cells11071213

**Published:** 2022-04-04

**Authors:** Kamila Filip, Anna Lewińska, Jagoda Adamczyk-Grochala, Antonella Marino Gammazza, Francesco Cappello, Marianna Lauricella, Maciej Wnuk

**Affiliations:** 1 Department of Biomedicine, Neurosciences and Advanced Diagnostics, Institute of Human Anatomy and Histology, University of Palermo, 90127 Palermo, Italy; kamila.filip.90@wp.pl (K.F.); antonella.marinogammazza@unipa.it (A.M.G.); francesco.cappello@unipa.it (F.C.); 2 Department of Biotechnology, Institute of Biology and Biotechnology, College of Nature Sciences, University of Rzeszow, 35959 Rzeszow, Poland; jadamczyk@ur.edu.pl (J.A.-G.); mwnuk@ur.edu.pl (M.W.); 3 Euro-Mediterranean Institutes of Science and Technology, 90139 Palermo, Italy; 4 Department of Biomedicine, Neurosciences and Advanced Diagnostics, Institute of Biochemistry, University of Palermo, 90127 Palermo, Italy

**Keywords:** 5-azacytidine, oxidative stress, insulinoma, Trdmt1, nitric oxide, autophagy

## Abstract

5-Azacytidine (5-azaC), a methyltransferase inhibitor and anticancer drug, can promote several cellular stress responses such as apoptosis, autophagy, and senescence. The action of 5-azaC is complex and can be modulated by dose, time of treatment, and co-administration with oxidants. Insulinoma is a rare pancreatic neuroendocrine tumor with limited chemotherapeutic options. In the present study, two cellular models of insulinoma were considered, namely NIT-1 and β-TC-6 mouse cells, to evaluate the effects of 5-azaC post-treatment during hydrogen peroxide-induced oxidative stress. 5-azaC attenuated the development of oxidant-induced senescent phenotype in both cell lines. No pro-apoptotic action of 5-azaC was observed in cells treated with the oxidant. On the contrary, 5-azaC stimulated an autophagic response, as demonstrated by the increase in phosphorylated eIF2α and elevated pools of autophagic marker LC3B in oxidant-treated β-TC-6 cells. Notably, autophagy resulted in increased necrotic cell death in β-TC-6 cells with higher levels of nitric oxide compared to less affected NIT-1 cells. In addition, 5-azaC increased levels of RNA methyltransferase Trdmt1, but lowered 5-mC and m^6^A levels, suggesting Trdmt1 inhibition. We postulate that the 5-azaC anticancer action may be potentiated during oxidative stress conditions that can be used to sensitize cancer cells, at least insulinoma cells, with limited drug responsiveness.

## 1. Introduction

Insulinoma pancreatic endocrine tumors are rare gastrointestinal neuroendocrine tumors (GI NET) that originate from islet β-cells and occur in one to four people per million per year in the general population [1]. Insulinoma β-cells are overproducers of insulin, so they promote hypoglycemia and, consequently, the development of symptoms of neuroglycopenia and the catecholaminergic response [2]. It is worth noting that insulinomas affect women more frequently than men [3] and are the most often diagnosed in people between the ages of 40 to 60 [3,4]. In approximately 90% of cases, pancreatic insulin-producing tumors are mild [5]. However, in some patients, they may develop metastatic disease several years after the excision of insulinomas [6,7]. Despite the availability of multimodal treatment options, including surgery, chemotherapy, embolization, radiofrequency ablation, somatostatin analogs, insulin secretion inhibitors, and mTOR or tyrosine kinase inhibitors, the prognosis of malignant insulinoma is poor and the median survival is approximately 2 years [8,9]. Chemotherapy-based treatment of malignant insulinomas relies on the co-administration of streptozotocin and doxorubicin and/or 5-fluorouracil (5-FU), which may reduce hormonal symptoms in 6 to 70% of metastatic insulinoma patients [8].

A side effect of chemotherapy may be stress-induced premature senescence (SIPS) observed in both treated cancer cells as well as normal adjacent cells [10,11]. Normal and neoplastic senescent cells can secrete numerous proinflammatory agents into their microenvironment (senescence-associated secretory phenotype, SASP) that can promote proliferation, migration, invasiveness of cancer cells, angiogenesis, epithelial-mesenchymal transition (EMT), and metastasis [12,13,14]. Effective strategies based on the elimination of the harmful effects of the presence of senescent cells (senotherapies) are currently being developed [12,13]. There are at least two experimental approaches to limit adverse side-effects of chemotherapy-induced senescence, namely selective elimination of senescent cells (senolysis) and attenuation of SASP (senostasis) [12,13]. Currently, more than thirty different compounds with senolytic activity have been identified such as BCL-2 family inhibitors, HSP90 inhibitors, p53 pathway targeting compounds, natural products and their analogs, cardiac glycosides, and many more [15]. However, potent senolytic action may be cell-type-specific and successful senotherapy may require combined treatment of senolytics such as natural compound quercetin and a kinase inhibitor dasatinib [15]. There are limited data on the identification of compounds that can attenuate the development of SIPS in normal and cancer cells (anti-senescence agents) when added before the senescence program was activated, and characterization of related cellular responses modulating cell fates.

It is widely accepted that type 2 diabetes (T2D) is an age-related disease, however, β-cell senescence is also implicated in the progression of type 1 diabetes (T1D) [16]. In the present study, two cellular models of insulinoma were used, namely NIT-1, a model of T1D, and β-TC-6 cells, a model of T2D, to analyze the potential pleiotropic effects of an epigenetic anticancer drug 5-azacytidine (5-azaC) during hydrogen peroxide (HP)-induced oxidative stress, such as anti-senescence, pro-apoptotic and pro-autophagic actions. The anti-senescence potential of 5-azaC was revealed in both cell lines treated with a concentration of HP that induces senescence. Furthermore, 5-azaC post-treatment promoted cytotoxic autophagy in oxidant-stimulated β-TC-6 cells that was accompanied by nitric oxide-mediated necrotic cell death and Trdmt1-based stress response. We showed that 5-azaC can promote the elimination of insulinoma cells stressed by an oxidant by sensitizing them to cell death mediated by nitric oxide. Thus, a new anticancer experimental approach is proposed.

## 2. Materials and Methods

### 2.1. Cell Lines and Culture Conditions

Insulinoma cell lines NIT-1 (CRL-2055™, derived from NOD/Lt-Tg(RIPTag)1Lt mouse strain, a model of T1D) and β-TC-6 (CRL-11506™, derived from (C57BL/6J × DBA/2J)F2 RIP1Tag2 mouse strain, a model of T2D) were established from transgenic mice for the SV40 large T antigen under the control of a rat insulin promoter and purchased from ATCC (Manassas, VA, USA). Cells were cultured at 37 °C in the presence of 5% CO_2_ in Dulbecco’s Modified Eagle’s Medium (DMEM with 4.5 g/L glucose, L-glutamine, and sodium pyruvate, 10-013-CV, Corning, Tewksbury, MA, USA) enriched with 15% FBS (Corning, Tewksbury, MA, USA, 35-079-CV) and antibiotic antimycotic solution (100 U/mL penicillin, 0.1 mg/mL streptomycin and 0.25 μg/mL amphotericin B) (Corning, Tewksbury, MA, USA, 30-004-Cl). Cells were passaged by detaching with the trypsin-EDTA solution (Corning, Tewksbury, MA, USA, 25-053-CI). Both cell lines were seeded at the total number of 1 × 10^5^ cells per well in a 6-well plate and cultured until approximately 70–80% confluency was reached (3 to 4 days) and then treated with 100 µM hydrogen peroxide (H_2_O_2_) for 24 h (HP, Merck KGaA, Darmstadt, Germany) for the induction of oxidative stress, or with 1 µM 5-azacitidine for 24 h (5-azaC, Merck KGaA, Darmstadt, Germany) for the inhibition of methylation or cells were treated with 100 µM HP for 24 h and then the medium was discarded and cells were post-treated with 1 µM 5-azaC for 24 h. The concentrations of HP and 5-azaC were selected on the basis of our previously published protocols [11,17]. Except for the assay of senescence-associated β-galactosidase activity, all parameters were analyzed immediately after 5-azaC post-treatment.

### 2.2. Cell Number

After HP and 5-azaC treatments, cell proliferation as a cell number was automatically analyzed using TC10™ Automated Cell Counter (Bio-Rad, Hercules, CA, USA).

### 2.3. Cell Cycle

For DNA content-based analysis of the cell cycle, imaging flow cytometry was used. After 5-azaC post-treatment, cells were fixed using 4% paraformaldehyde (PFA) at room temperature for 20 min, washed in DPBS, resuspended in 70% ethanol, and incubated on ice for 20 min. DNA was visualized using propidium iodide (PI) staining. The subpopulations of cells at the phases G0/G1, S, and G2/M of the cell cycle were analyzed using Amnis^®^ FlowSight^®^ imaging flow cytometer and IDEAS software version 6.2.187.0 (Luminex Corporation, Austin, TX, USA).

### 2.4. Senescence-Associated β-Galactosidase Activity (SA-β-Gal)

Insulinoma cells were treated with 100 µM HP for 24 h and then post-treated with 1 µM 5-azaC for 24 h and cultured up to 7 days after drug removal. Insulinoma cells were then fixed using a fixation solution (2% formaldehyde and 0.2% glutaraldehyde in DPBS, Merck KGaA, Darmstadt, Germany), then washed in DPBS and incubated overnight at 37 °C with a solution containing 1 mg/mL 5-bromo-4-chloro-3-indolyl-β-D-galactopyranoside, 5 mM potassium ferrocyanide, 5 mM potassium ferricyanide, 150 mM NaCl, 2 mM MgCl_2_ in 40 mM citrate phosphate buffer, pH 6.0 (Merck KGaA, Darmstadt, Germany) [18]. The cells were then assayed using an Olympus BX71 light inverted microscope with a DP72 CCD camera and an analysis system CellB (Shinjuku, Tokyo, Japan). The intensity of SA-β-gal-positive signals (blue signals) was evaluated using ImageJ software (https://imagej.nih.gov/ij/, accessed on 18 March 2022, version 1.53k). SA-β-gal-positive signals under untreated conditions were considered as 1.0 for each cell line and treated samples were normalized to corresponding control samples.

### 2.5. Apoptosis

After HP and 5-azaC treatments, phosphatidylserine externalization (an apoptotic biomarker) was analyzed using a Muse^®^ Cell Analyzer and Muse^®^ Annexin V and Dead Cell Assay Kit (Luminex Corporation, Austin, TX, USA) as described elsewhere [11].

### 2.6. Oxidative Stress Parameters

After HP and 5-azaC treatments, selected parameters of oxidative stress were considered such as superoxide levels, glutathione redox potential, and oxidative DNA damage. Superoxide levels were evaluated using Muse^®^ Cell Analyzer and Muse^®^ Oxidative Stress Kit (Luminex Corporation, Austin, TX, USA) as previously described [19]. Briefly, superoxide-positive and superoxide-negative subpopulations were automatically analyzed and representative histograms are shown. The glutathione redox potential (GSH/GSSG) was assayed using a Premo™ Cellular Redox Sensor (roGFP-Grx1, P36242, Thermo Fisher Scientific, Waltham, MA, USA) as described comprehensively elsewhere [19]. Briefly, a baculovirus-based transduction (BacMam 2.0 technology) was used to provide a redox-sensitive green fluorescent protein (roGFP) and glutaredoxin 1 (Grx1) chimeric protein into insulinoma cells and glutathione redox potential-based fluorescent signals (relative fluorescent units, RFU) were measured using a Tecan Infinite^®^ M200 (Tecan Group Ltd., Männedorf, Switzerland) fluorescence mode microplate reader. The GSH/GSSG results were calculated as a ratio of RFU_400 nm_ to RFU_488 nm_. The levels of 8-hydroxy-2′-deoxyguanosine (8-oxo-dG) in total DNA were measured using EpiQuik 8-OHdG DNA Damage Quantification Direct Kit (Epigentek, Farmingdale, NY, USA) as previously described [20]. DNA oxidative damage at control conditions was considered as 1.0 and data were normalized to control (Ctrl).

### 2.7. Nitric Oxide Levels

After HP and 5-azaC treatments, nitric oxide levels were analyzed using Muse^®^ Cell Analyzer and Muse^®^ Nitric Oxide Kit (Luminex Corporation, Austin, TX, USA) as previously described [21]. Briefly, nitric oxide specific fluorogenic probe DAX-J2^TM^ orange and a death marker, 7-aminoactinomycin D (7-AAD) were used to discriminate between the production of nitric oxide in live (7-AAD-negative) and dead (7-AAD-positive) cells. Flow cytometry measurements were automatically performed and representative dot plots are presented.

### 2.8. RNA Methylation

After HP and 5-azaC treatments, two parameters of RNA methylation were evaluated, namely the levels of 5-methylcytosine (5-mC) and N^6^-methyladenosine (m^6^A). Dedicated ELISA-based assays MethylFlash 5-mC RNA Methylation ELISA Easy Kit and EpiQuik m^6^A RNA Methylation Quantification Kit, respectively, were used (EpiGentek, Farmingdale, NY, USA) as previously described [17].

### 2.9. Immunofluorescence Using Imaging Flow Cytometry

After HP and 5-azaC treatments, insulinoma cells were fixed as described above (cell cycle subsection). Fixed cells were then incubated with blocking solution (1% BSA in DPBS) at room temperature for 30 min. After blocking, cells were incubated with the following primary antibodies: anti-Dnmt2/Trdmt1 (D-9, sc-365001, 1:100, Santa Cruz Biotechnology, Dallas, TX, USA), LC3B antibody (2775, 1:100, Cell Signaling Technology, Danvers, MA, USA), and anti-phospho-eIF2α Ser52 (44728G, 1:100, Thermo Fisher Scientific, Waltham, MA, USA) and dedicated fluorochrome conjugated secondary anti-mouse and anti-rabbit antibodies (A32723, A32731, A10523, 1:500, Thermo Fisher Scientific, Waltham, MA, USA). DNA was visualized using PI staining. Fluorescent immuno-signals were captured using Amnis^®^ FlowSight^®^ imaging flow cytometer (Luminex Corporation, Austin, TX, USA). To evaluate the levels of Trdmt1, phospho-eIF2α, and LC3B, and analyze the cell subpopulation with dual Trdmt1 and phospho-eIF2α signals, IDEAS software version 6.2.187.0 (Luminex Corporation, Austin, TX, USA) was used.

### 2.10. Statistical Analysis

The results were calculated as the mean ± SD from at least three independent experiments. Differences between control conditions (Ctrl) and treated samples (HP, 5-azaC and 5-azaC post-treatment) were evaluated using one-way ANOVA and Dunnett’s a posteriori test, and differences between HP treatment and 5-azaC post-treatment were evaluated using one-way ANOVA and Tukey’s a posteriori test. Statistical significance was analyzed using GraphPad Prism 5. *p*-values of less than 0.05 were considered significant.

## 3. Results and Discussion

### 3.1. 5-AzaC Attenuates the Development of HP-Induced Senescent Phenotype in Insulinoma Cells

**Figure 1:**
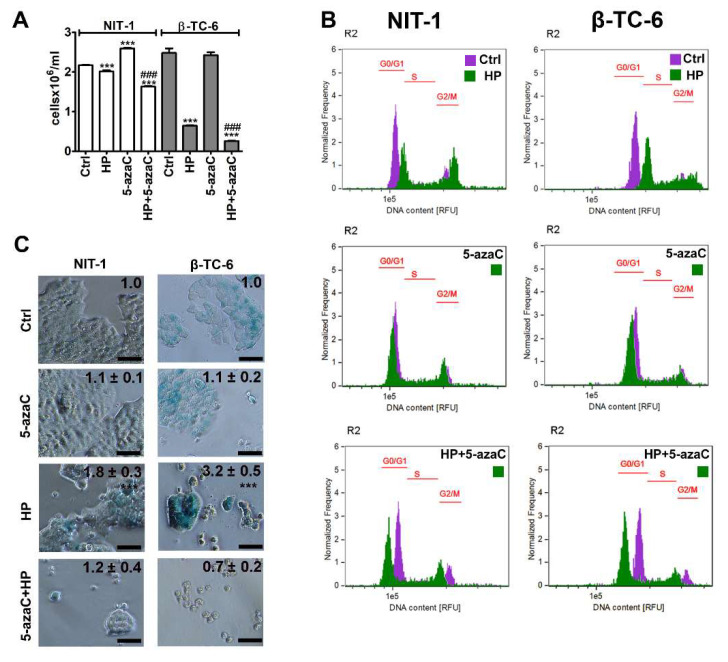
5-AzaC post-treatment-mediated changes in cell number (**A**), cell cycle (**B**) and senescence-associated β-galactosidase (SA-β-gal) activity (**C**) in hydrogen peroxide-treated mouse insulinoma NIT-1 and β-TC-6 cells. Insulinoma cells were stimulated with 100 µM HP for 24 h and then post-treated with 1 µM 5-azaC for 24 h. (**A**) Cell number was calculated automatically using dedicated cell counter. Bars indicate SD, n = 3, *** *p* < 0.001 compared to Ctrl (ANOVA and Dunnett’s a posteriori test), ^###^ *p* < 0.001 compared to HP-treated cells (ANOVA and Tukey’s a posteriori test). (**B**) DNA content-based analysis of cell cycle was conducted using imaging flow cytometry. Representative histograms are shown. Violet, untreated samples; green, treated samples. The phases of cell cycle (G0/G1, S and G2/M) are denoted using red horizontal lines. (**C**) SA-β-gal activity was assayed after 7 days of 5-azaC removal using dedicated biochemical test. Representative microphotographs are shown. Objective 20×. Quantitative analysis of the intensity of SA-beta-gal-positive signals (blue signals) is also provided. SA-beta-gal-positive signals at untreated conditions were considered as 1.0 for each cell line and treated samples were normalized to corresponding control samples, n = 3, *** *p* < 0.001 compared to Ctrl (ANOVA and Dunnett’s a posteriori test). Ctrl, control conditions; HP, hydrogen peroxide (H_2_O_2_) treatment; 5-azaC, azacytidine treatment; HP+5-azaC, hydrogen peroxide treatment, and azacytidine post-treatment.

To evaluate the effects of a methyltransferase inhibitor and an epigenetic anticancer drug 5-azaC against insulinoma cells during stress conditions, the HP-induced oxidative stress approach was used [17]. We have previously shown that 2 h treatment with 100 µM HP promoted SIPS in normal human fibroblasts such as WI-38 and BJ cells that were accompanied by elevated levels of reactive oxygen species (ROS) [17]. In the present study, the same concentration of HP was used, but insulinoma cells were stimulated with HP for 24 h. 5-AzaC post-treatment (1 µM, 24 h) was applied as previously reported using a doxorubicin- and etoposide-induced senescence model using four genetically different cancer cell lines [11]. HP treatment reduced the cell number of both insulinoma cell lines, but the cytostatic effects of HP were more pronounced in β-TC-6 cells compared to NIT-1 cells (Figure 1A). HP lowered the cell number to 93% and 26% of control levels in NIT-1 and β-TC-6 cells, respectively, (*p* < 0.001, Figure 1A). 5-AzaC alone had no effect on β-TC-6 cells but increased the number of NIT-1 cells (*p* < 0.001, Figure 1A). 5-AzaC post-treatment potentiated HP-induced reduction of cell number in both cell lines (*p* < 0.001, Figure 1A). Cell cycle analysis (Figure 1B) demonstrated that HP promoted cell cycle arrest at the G2/M phase in both cell lines (Figure 1B). 5-AzaC alone had no effect on cell cycle progression in insulinoma cells (Figure 1B) while 5-azaC post-treatment suppressed HP-induced G2/M phase cell cycle arrest in NIT-1 and β-TC-6 cells (Figure 1B). We were then interested if HP cytostatic effects were transient or permanent and also associated with SIPS in insulinoma cells and how 5-azaC may modulate the activation of the SIPS program. Thus, senescence-associated β-galactosidase (SA-β-gal) activity was analyzed and we found that HP also induced SIPS in two insulinoma cell lines (Figure 1C). No pro-senescent activity of 5-azaC was observed (Figure 1C). On the contrary, 5-azaC post-treatment inhibited the activation of the HP-mediated senescence program in NIT-1 and β-TC-6 cells as judged by the limited number of SA-β-gal-positive cells after 7 days of drug removal (Figure 1C).

We did not analyze the effect of 5-azaC in HP-induced senescent insulinoma cells as 5-azaC was added before the development of the senescent phenotype. Thus, one cannot conclude if 5-azaC may also have senolytic or senostatic activity in our experimental settings. However, we have previously documented the senolytic activity of 5-azaC in chemotherapy-induced senescent cancer cells lacking the active RNA methyltransferase gene (*DNMT2*/*TRDMT1* gene) [11]. *TRDMT1* gene KO potentiated 5-azaC-mediated apoptosis-based senolysis in doxorubicin- and etoposide-induced senescent HeLa cervical cancer cells and 5-azaC-mediated necrosis-based senolysis in etoposide-induced senescent U-2 OS osteosarcoma cells [11]. Thus, in certain experimental conditions, senolytic activity of 5-azaC can be revealed that can be based on different modes of cell death such as apoptosis or necrosis [11]. 5-AzaC and its deoxy derivative 5-aza-2′-deoxycytidine (5-azadC, decitabine) are inhibitors of DNA methylation, but only 5-azaC is capable to inhibit cytosine 38 methylation of tRNA^Asp^ that is a substrate of DNMT2/TRDMT1 [22]. It has been postulated that they can stimulate diverse responses in cancer cells, for example, 5-azaC can promote apoptosis, whereas 5-azadC can induce cellular senescence [23]. In human colorectal cancer cells, 5-azaC induced apoptosis and concurrently promoted cytoprotective autophagy, whereas 5-azadC caused cell cycle arrest at the G2/M phase associated with p53 induction [24]. However, their molecular action might not be limited to one type of cellular response. For example, differentiation, senescence, and autophagy in 5-azadC-treated chronic myeloid leukemia (CML) are important cellular events enabling cell sensitization and 5-azadC-mediated apoptotic cell death [25]. 

### 3.2. 5-AzaC Potentiates Nitric Oxide-Mediated Necrotic Cell Death in HP-Treated β-TC-6 Cells

**Figure 2:**
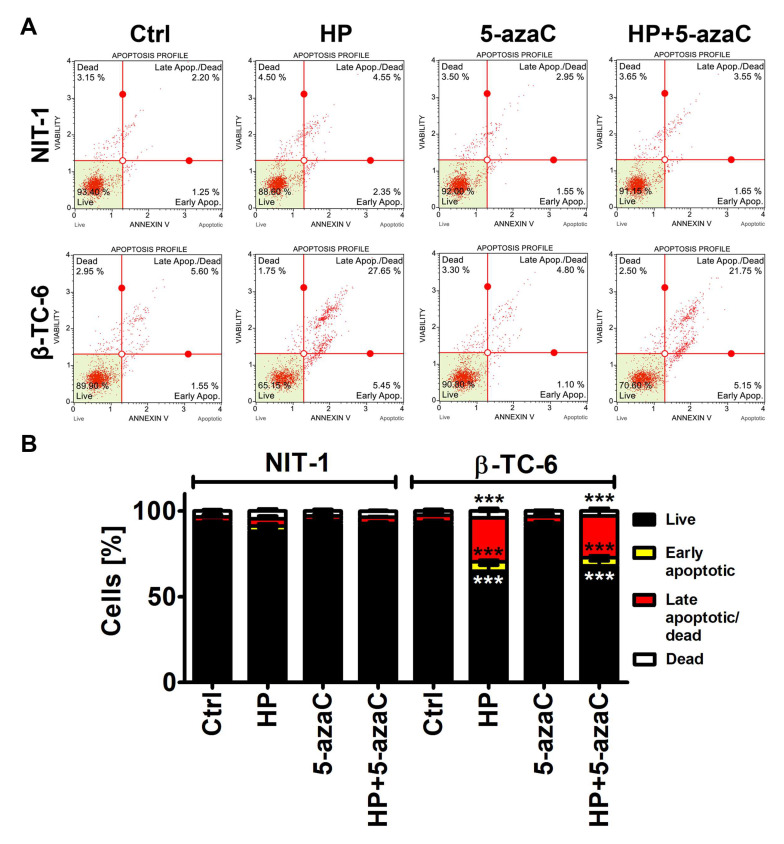
5-AzaC post-treatment-mediated apoptosis in hydrogen peroxide-treated mouse insulinoma NIT-1 and β-TC-6 cells. Insulinoma cells were stimulated with 100 µM HP for 24 h and then post-treated with 1 µM 5-azaC for 24 h. Apoptosis was evaluated using Annexin V staining and flow cytometry. (**A**) Representative dot plots are shown. (**B**) Bars indicate SD, n = 3, *** *p* < 0.001 compared to Ctrl (ANOVA and Dunnett’s a posteriori test). Ctrl, control conditions; HP, hydrogen peroxide (H_2_O_2_) treatment; 5-azaC, azacytidine treatment; HP+5-azaC, hydrogen peroxide treatment, and azacytidine post-treatment.

We were then interested if a more potent decrease in the cell count in HP-treated β-TC-6 cells compared to HP-treated NIT-1 cells may be associated with increased cytotoxicity as well. Indeed, HP treatment resulted in apoptotic cell death in β-TC-6 cells (*p* < 0.001, Figure 2B), whereas slight and insignificant changes in phosphatidylserine externalization were noticed in HP-treated NIT-1 cells.

5-AzaC alone was not cytotoxic against both cell lines and 5-azaC post-treatment did not augment HP-induced apoptosis in β-TC-6 cells (Figure 2).

**Figure 3:**
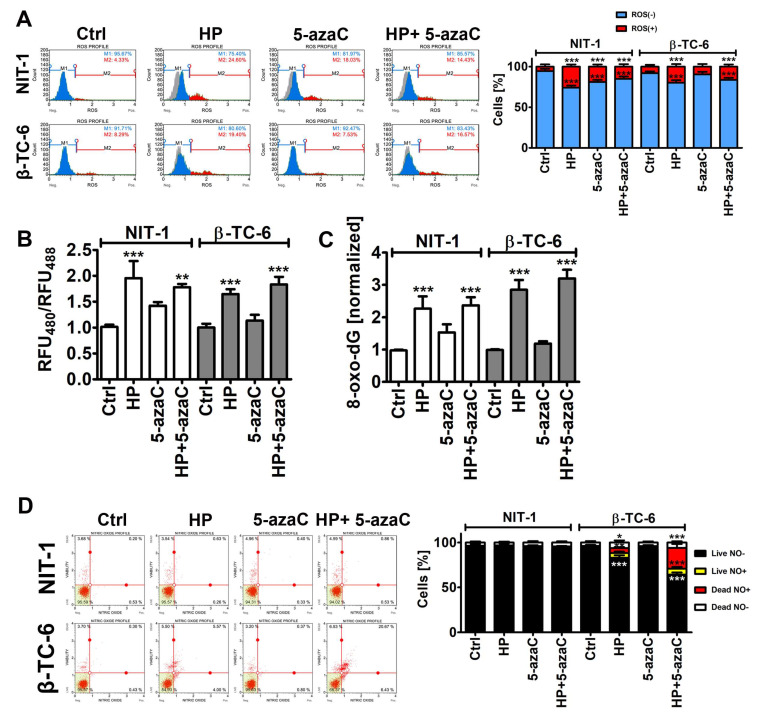
5-AzaC post-treatment-mediated oxidative stress (**A**,**B**,**C**) and nitrosative stress (**D**) in hydrogen peroxide-treated mouse insulinoma NIT-1 and β-TC-6 cells. Insulinoma cells were stimulated with 100 µM HP for 24 h and then post-treated with 1 µM 5-azaC for 24 h. (**A**) Superoxide levels were analyzed using dihydroethidium (DHE) staining and flow cytometry. Representative histograms are shown. A gray control histogram (Ctrl) is overlayed on each sample. Two subpopulations were considered and quantified [%], namely superoxide-negative subpopulation (blue) and superoxide-positive subpopulation (red). Bars indicate SD, n = 3, *** *p* < 0.001 compared to Ctrl (ANOVA and Dunnett’s a posteriori test). (**B**) The glutathione redox potential was analyzed using roGFP-Grx1 redox sensor according to the manufacturer’s instructions. The data are presented as a ratio of fluorescent measurements (relative fluorescent unit, RFU) at 400 nm and 488 nm. Bars indicate SD, n = 3, *** *p* < 0.001, ** *p* < 0.01 compared to Ctrl (ANOVA and Dunnett’s a posteriori test). (**C**) The levels of 8-hydroxy-2′-deoxyguanosine (8-oxo-dG) in total DNA were measured using dedicated enzyme-linked immunosorbent assay (ELISA). The data were normalized to Ctrl. Bars indicate SD, n = 3, *** *p* < 0.001 compared to Ctrl (ANOVA and Dunnett’s a posteriori test). (**D**) Nitric oxide levels were analyzed using DAX-J2^TM^ orange staining and flow cytometry. Bars indicate SD, n = 3, *** *p* < 0.001, ** *p* < 0.01, * *p* < 0.05 compared to Ctrl (ANOVA and Dunnett’s a posteriori test). Representative dot plots are shown. Ctrl, control conditions; HP, hydrogen peroxide (H_2_O_2_) treatment; 5-azaC, azacytidine treatment; HP+5-azaC, hydrogen peroxide treatment, and azacytidine post-treatment.

As 5-azaC and 5-azadC can exert their anticancer effects through oxidative stress [26,27], we have then examined the effects of 5-azaC post-treatment on selected parameters of oxidative stress (Figure 3).

Of course, HP treatment caused an increase in the levels of superoxide, affected glutathione redox potential, and promoted oxidative DNA damage in both insulinoma cell lines (Figure 3A–C). However, increased cytotoxicity (apoptosis induction) in HP-treated β-TC-6 cells (Figure 2) was not accompanied by increased oxidative stress compared to HP-treated NIT-1 cells (Figure 3A–C). Except for the 5-azaC-mediated increase in the levels of superoxide in NIT-1 cells, 5-azaC alone did not affect the parameters of oxidative stress (Figure 3A–C). A 24 h treatment with 1 µM 5-azaC also did not augment superoxide levels in four different cancer cell lines (HeLa, MDA-MB-231, U-2 OS and U-251 MG cells) [11]. 5-AzaC post-treatment did not intensify oxidative stress in HP-treated insulinoma cells (Figure 3A–C). It has been reported that 5-azaC promoted oxidative stress and apoptosis when co-treated with MS-275, a histone deacetylase inhibitor (HDACi), in leukemia cells [28]. However, synergistic cytotoxicity and apoptosis were observed when two drugs were applied at the concentration of 5 µM, which is a five-time higher concentration than used in the present study [28]. Furthermore, 5-azadC, a derivative of 5-azaC, when co-treated with paracetamol, promoted glutathione depletion and ROS accumulation that had an anticancer action against head and neck squamous cell carcinoma and acute myeloid leukemia cell lines [29]. The sensitizing effect of paracetamol supplementation was not achieved when 5-azaC was co-administered [29]. Thus, oxidative stress-mediated anticancer action of combined treatment is specific to 5-azadC [29]. On the contrary, 5-azaC post-treatment augmented the levels of nitric oxide in the necrotic cell population (nitric oxide-positive and 7-AAD-positive cells) compared to HP-treated β-TC-6 cells (Figure 3D). Nitric oxide production was increased by about 15% after 5-azaC post-treatment compared to HP stimulation in β-TC-6 cells (Figure 3D). Similar effects were not observed in NIT-1 cells (Figure 3D). Moreover, HP treatment in β-TC-6 cells resulted in a decrease in the live nitric oxide-negative cell population of about 10% compared to HP-treated NIT-1 cells (Figure 3D). Thus, one can conclude that the more pronounced effect of 5-azaC post-treatment in β-TC-6 cells is associated with nitric oxide-mediated necrotic cell death compared to less affected NIT-1 cells (Figure 3D). According to our knowledge, there are no data on 5-azaC-mediated changes in intracellular nitric oxide levels and their biological consequences in cancer cells. The effects of 5-azadC on nitric oxide synthesis can be contradictory depending on cell type [30,31]. 5-AzadC promoted proinflammatory cytokine (IFN-γ, TNF-α, IL-1)-mediated nitric oxide production in C6 rat astrocytoma cell line and primary astrocytes, but not in IFN-γ-stimulated macrophages, fibroblasts, or endothelial cells [30]. The authors concluded that 5-azadC-mediated elevation in nitric oxide production was achieved by increased iNOS gene expression rather than changes in its catalytic activity and p44/42 MAPK signaling was involved in 5-azadC action [30]. On the contrary, co-treatment of 5-azadC with paclitaxel suppressed IFN-γ-induced nitric oxide release from mouse L929 fibrosarcoma cells and primary fibroblasts that was due to decreased expression in iNOS [31]. Thus, 5-azadC may modulate nitric oxide production depending on experimental settings involving different co-stimulators [30,31].

### 3.3. Trdmt1 Is Increased after HP and 5-azaC Post-Treatment in β-TC-6 Cells

**Figure 4:**
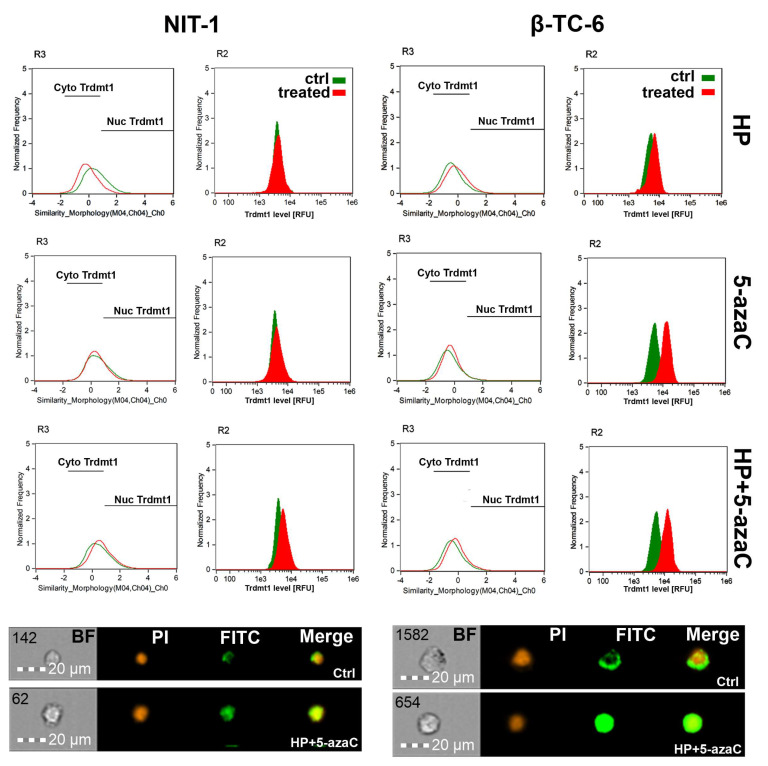
5-AzaC post-treatment-mediated changes in the levels and intracellular localization of Trdmt1 in hydrogen peroxide-treated mouse insulinoma NIT-1 (**left**) and β-TC-6 cells (**right**). Insulinoma cells were stimulated with 100 µM HP for 24 h and then post-treated with 1 µM 5-azaC for 24 h. To analyze Trdmt1 signals, immunofluorescence and imaging flow cytometry were used. Representative histograms are shown (**top**). Green histograms indicate untreated samples and red histograms indicate treated samples. Cytoplasmic (Cyto) and nuclear (Nuc) signals of Trdmt1 are denoted using black horizontal lines. Representative images are also shown (**bottom**). BF, bright field; PI, nucleus staining using propidium iodide staining (red); FITC, immunostaining of Trdmt1 using secondary antibody conjugated to Alexa Fluor Plus 488 (green); Merge, a merged image of PI and FITC. Ctrl, control conditions; HP, hydrogen peroxide (H_2_O_2_) treatment; 5-azaC, azacytidine treatment; HP+5-azaC, hydrogen peroxide treatment, and azacytidine post-treatment.

TRDMT1 (tRNA aspartic acid methyltransferase 1, formerly known as DNA methyltransferase 2, DNMT2), is involved in the maintenance of tRNA stability and translation via RNA C-5 methylation [32,33,34,35]. We have previously reported that during oxidative stress conditions, mouse Trdmt1 and human TRDMT1 can modulate cellular responses associated with cell proliferation, survival, and senescence using fibroblast models in vitro [17,36]. TRDMT1 is also documented to be an important regulator of cancer cell proliferation, migration, and DNA damage response (DDR) [37,38,39]. Indeed, the knockout of the *TRDMT1* gene prevented drug resistance in osteosarcoma cells [38]. Thus, targeting TRDMT1 is postulated as a novel anticancer strategy [38]. In the present study, we have analyzed the changes in the levels of Trdmt1 and its cellular localization upon HP stimulation and after 5-azaC post-treatment, as 5-azaC can serve as a TRDMT1/Trdmt1 inhibitor [22]. The levels of Trdmt1 in HP-treated NIT-1 cells were almost unchanged and only 5-azaC post-treatment resulted in a moderate increase in Trdmt1 pools and nuclear translocation of Trdmt1 (Figure 4).

On the contrary, HP stimulation in β-TC-6 cells promoted an increase in the levels of Trdmt1 that was potentiated after 5-azaC post-treatment (Figure 4). 5-AzaC alone also caused elevated levels of Trdmt1 in β-TC-6 cells (Figure 4). All treatments in β-TC-6 cells also resulted in Trdmt1 nuclear translocation (Figure 4). Predominant cellular localization of Trdmt1 and its changes during stress conditions are still a matter of debate [32,40]. The GFP-TRDMT1 fusion protein can be found predominantly in the cytoplasm, whereas endogenous TRDMT1 is localized in the nucleus in mammalian cells [40]. Human TRDMT1 protein was also shown to be primarily localized to the cytoplasm of transfected mouse fibroblasts [32]. Under stress conditions, TRDMT1 can dynamically shuttle between the nucleus and the cytoplasm [40]. It was documented that TRDMT1 is located in cytoplasmic stress granules and RNA processing bodies in stressed cells [40]. The cytoplasmic pool of Trdmt1 was also increased after HP stimulation in NIT-1 cells, but not in HP-treated β-TC-6 cells (Figure 4). However, one can speculate that elevated levels of Trdmt1 cannot be a part of an adaptive stress response in β-TC-6 cells as 5-azaC post-treatment can inhibit the activity of Trdmt1 that can, in turn, sensitize β-TC-6 cells to nitric oxide-mediated necrotic cell death (Figure 3D).

### 3.4. 5-AzaC Stimulation Promotes Cytotoxic Autophagy in HP-Stressed β-TC-6 Cells

**Figure 5:**
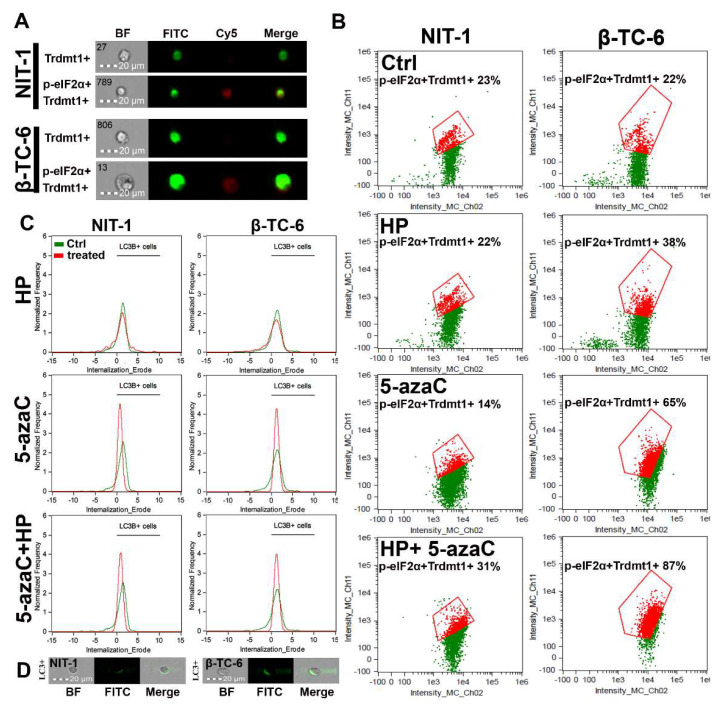
5-AzaC post-treatment-mediated changes in Trdmt1 and p-eIF2α signals (**A**,**B**) and autophagic marker LC3B (**C**,**D**) in hydrogen peroxide-treated mouse insulinoma NIT-1 and β-TC-6 cells. Insulinoma cells were stimulated with 100 µM HP for 24 h and then post-treated with 1 µM 5-azaC for 24 h. To analyze cellular subpopulation characterized by dual positive signals of Trdmt1 and p-eIF2α, immunofluorescence and imaging flow cytometry were used. (**A**) Representative images are shown. BF, bright field; FITC, immunostaining of Trdmt1 using secondary antibody conjugated to Alexa Fluor Plus 488 (green); Cy5, immunostaining of p-eIF2α using secondary antibody conjugated to Cyanine 5 (red); Merge, a merged image of FITC and Cy5. (**B**) Representative dot plots are also presented. Trdmt1-positive and p-eIF2α-positive subpopulations are indicated in red and quantified [%]. (**C**,**D**) To analyze LC3B signals, immunofluorescence and imaging flow cytometry were used. (**C**) Representative histograms are shown (green, untreated samples; red, treated samples). Black horizontal lines are provided to emphasize LC3B-positive cell populations. (**D**) Representative images are also shown. BF, bright field; FITC, immunostaining of LC3B using secondary antibody conjugated to Alexa Fluor Plus 488 (green); Merge, a merged image of BF and FITC. Ctrl, control conditions; HP, hydrogen peroxide (H_2_O_2_) treatment; 5-azaC, azacytidine treatment; HP+5-azaC, hydrogen peroxide treatment, and azacytidine post-treatment.

Autophagy is a physiological self-degradative process that is activated in different cell stress conditions to eliminate misfolded proteins or damaged subcellular organelles through lysosomes [41,42]. Autophagy is considered an important survival mechanism; however, it may also mediate cell death during elevated cellular stress [41,42]. Autophagy may be implicated in different fates of cancer cells during chemotherapy [41,42]. For example, autophagy may protect against drug-induced apoptosis or may potentiate cytotoxic effects of chemotherapeutic agents having both cytoprotective and cytotoxic functions [41,42]. 5-AzaC caused an accumulation of LC3B, a marker of autophagy, and LC3B silencing increased cell death and caspase 3 activity in SKM1 myeloid cells [43]. Thus, autophagy is considered a protective mechanism in 5-azaC-treated SKM1 cells [43]. 5-AzaC also promoted an acute apoptotic response that was antagonized by concurrently induced cytoprotective autophagy in human colorectal cancer cells [24]. On the contrary, autophagy is required for 5-azadC-mediated apoptotic cell death in K-562 and MEG-01 CML cells [25]. Under different kinds of cellular stress, eukaryotic initiation factor-2α (eIF2α) undergoes phosphorylation. This phosphorylation event is important for the induction of autophagy [44]. As phosphorylation of eIF2α is implicated in the induction of drug-mediated autophagy in different experimental settings [44], we have then investigated if 5-azaC post-treatment may result in both Trdmt1 induction (Figure 4) and phosphorylation of eIF2α (p-eIF2α) (Figure 5A,B). We have analyzed a cell population with dual signals of Trdmt1 and p-eIF2α and found that 5-azaC post-treatment caused an increase in Trdmt1-positive and p-eIF2α-positive populations in both NIT-1 and β-TC-6 cells (Figure 5B). This effect was more pronounced in β-TC-6 cells than in NIT-1 cells (an increase of 8% and 65%, respectively) (Figure 5B). Moreover, 5-azaC alone also promoted an increase in dual signals of Trdmt1 and p-eIF2α in β-TC-6 cells (Figure 5B). A similar effect was not observed in 5-azaC-treated NIT-1 cells (Figure 5B). We have also analyzed the levels of LC3B, a marker of autophagy during oxidative stress and after 5-azaC post-treatment (Figure 5C,D). HP did not promote autophagy in both cell lines (Figure 5C). On the contrary, elevated signals of LC3B were observed after 5-azaC stimulation and 5-azaC post-treatment in NIT-1 and β-TC-6 cells (Figure 5C). As 5-azaC post-treatment in β-TC-6 cells resulted in nitric oxide-mediated necrotic cell death, one can speculate that accompanying autophagy may have a cytotoxic function in these particular conditions (Figure 5C).

### 3.5. HP-Induced Oxidative Stress Promotes RNA Methylation in Insulinoma Cells

**Figure 6:**
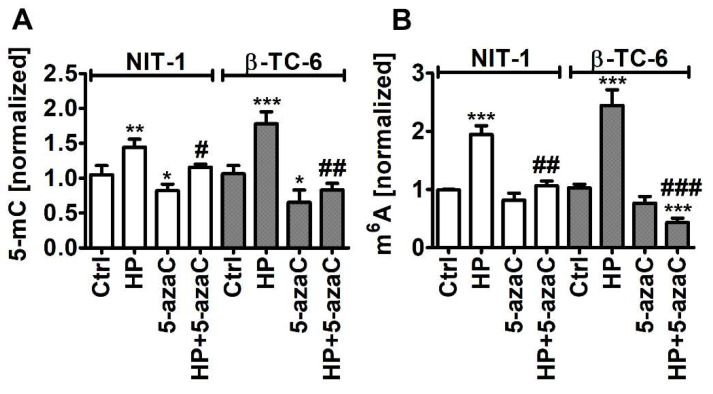
5-AzaC post-treatment-mediated changes in the levels of 5-methylcytosine (5-mC) (**A**) and N^6^-methyladenosine (m^6^A) (**B**) in RNA in hydrogen peroxide-treated mouse insulinoma NIT-1 and β-TC-6 cells. Insulinoma cells were stimulated with 100 µM HP for 24 h and then post-treated with 1 µM 5-azaC for 24 h. The levels of 5-mC and m^6^A were investigated using dedicated enzyme-linked immunosorbent assay (ELISA). The levels of 5-mC and m^6^A were normalized to Ctrl. Bars indicate SD, n = 3, *** *p* < 0.001, ** *p* < 0.01, * *p* < 0.05 compared to Ctrl (ANOVA and Dunnett’s a posteriori test), ^###^ *p* < 0.001, ^##^ *p* < 0.01, ^#^ *p* < 0.05 compared to HP-treated cells (ANOVA and Tukey’s a posteriori test). Ctrl, control conditions; HP, hydrogen peroxide (H_2_O_2_) treatment; 5-azaC, azacytidine treatment; HP+5-azaC, hydrogen peroxide treatment and azacytidine post-treatment.

As HP promoted Trdmt1 levels in β-TC-6 cells (Figure 4) and 5-azaC can be considered as an inhibitor of Trdmt1 [22], we have decided then to analyze the levels of 5-methylcytosine (5-mC) in total RNA during oxidative stress conditions and after 5-azaC post-treatment (Figure 6A).

Indeed, 5-azaC decreased the levels of 5-mC in both insulinoma cell lines (*p* < 0.05, Figure 6A). Oxidative stress promoted the levels of 5-mC that were more pronounced in HP-treated β-TC-6 cells (*p* < 0.001, Figure 6A) with elevated levels of Trdmt1 compared to HP-treated NIT-1 cells (Figure 4). 5-AzaC post-treatment decreased the levels of 5-mC compared to HP-treated treated β-TC-6 cells (*p* < 0.01, Figure 6A). This may suggest that despite the fact that 5-azaC post-treatment potentiated the levels of Trdmt1 compared to HP treatment in β-TC-6 cells (Figure 4), elevated pools of Trdmt1 are characterized by lower RNA methyltransferase activity due to the inhibitory action of 5-azaC (*p* < 0.01, Figure 6A). We have previously shown that HP stimulated the levels of 5-mC in total RNA in WI-38 human fibroblasts and this effect was not observed when Trdmt1 was silenced [17]. Of course, 5-azaC may also reduce the activity of other methyltransferases, which requires further studies.

As HP can also promote the levels of RNA N^6^-methyladenosine (m^6^A) in normal human fibroblasts (BJ cells) [17], we decided then to analyze the pools of m^6^A after HP stimulation and 5-azaC post-treatment (Figure 6B). Indeed, HP treatment resulted in an increase in the levels of m^6^A in both insulinoma cell lines (*p* < 0.001, Figure 6B). 5-AzaC post-treatment decreased the levels of m^6^A compared to HP stimulation in NIT-1 and β-TC-6 cells (*p* < 0.01 and *p* < 0.001, respectively, Figure 6B). Moreover, 5-azaC post-treatment diminished the pools of m^6^A compared to untreated control β-TC-6 cells (*p* < 0.001, Figure 6B). N^6^-methyladenosine, the most abundant modification on mRNAs in eukaryotes, is a methylation event that occurs in the N^6^-position of adenosine that is regulated by m^6^A methyltransferases (writers), m^6^A demethylases (erasers), and binding proteins (readers) [45,46,47]. The m^6^A motifs can affect RNA metabolism by the modulation of translation, splicing, export, degradation, and microRNA processing that is implicated in the regulation of physiological functions as well as pathological processes such as cancer [45,46,47]. The role of m^6^A during carcinogenesis is rather complex as both cancer-promoting (induction of oncogene expression and inhibition of tumor suppressor gene expression) and cancer-suppressing (inhibition of oncogene expression and induction of tumor suppressor gene expression) functions of m^6^A were postulated [45,46,47]. M^6^A RNA modification can also control autophagy by targeting selected autophagy-associated genes such as *ULK1*, *ATG5*, and *ATG7* [48,49]. The levels of m^6^A can be negatively correlated with autophagy as m^6^A-mediated degradation of *ULK1*, *ATG5* and *ATG7* genes suppressed autophagy [48,49]. On the contrary, the activity of FTO, m^6^A demethylase, can be positively correlated with autophagy as FTO reversed the m^6^A mRNA modification of autophagy-associated transcripts, thus preventing their degradation [48,49]. Decreased m^6^A signals were also associated with autophagy induction in our experimental setting, namely 5-azaC post-treatment resulted in diminished pools of m^6^A and elevated levels of LC3B compared to HP stimulation (Figure 5C and Figure 6B). M^6^A RNA modification can also modulate autophagy-mediated anticancer drug resistance [50,51]. Depletion of METTL3, a primary m^6^A methyltransferase, promoted sorafenib resistance in hepatocellular carcinoma (HCC) cells and activated autophagy-associated pathways by limited stability of FOXO3 mRNA under hypoxic conditions [52]. Overexpression of FOXO3 rescued the m^6^A-dependent sorafenib sensitivity by inhibiting autophagy [52]. METTL3-mediated autophagy also promoted gefitinib resistance in non-small cell lung cancer (NSCLC) cells, which was reversed by β-elemene treatment [53]. METTL3 positively regulated autophagy by increasing the levels of ATG5 and ATG7 and β-elemene, which prevented gefitinib resistance by inducing apoptosis and inhibiting autophagy [53]. Thus, in different experimental settings, the modulation of the m^6^A-mediated anticancer drug resistance may attenuate cytoprotective autophagy and promote cytotoxicity [52,53]. However, METTL3-associated drug resistance can be complex as METTL3 can be differently expressed in cancer samples [52,53]. For example, METTL3 is significantly down-regulated in human sorafenib-resistant hepatocellular carcinoma [52], whereas METTL3 is highly expressed in lung adenocarcinoma that is associated with gefitinib resistance of NSCLC cells [53]. In the present study, 5-azaC post-treatment also resulted in decreased levels of m^6^A (Figure 6B) that was accompanied by nitric oxide-mediated necrotic cell death and autophagy in β-TC-6 cells (Figure 3D and Figure 5C). We postulate that 5-azaC post-treatment-induced autophagy may have a cytotoxic function in β-TC-6 cells (Figure 3D and Figure 5C).

## 4. Conclusions

**Figure 7:**
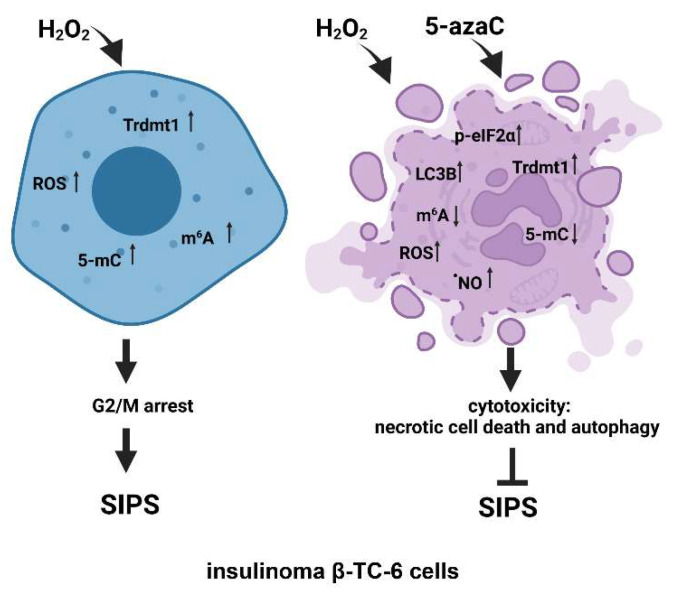
The effects of a methyltransferase inhibitor 5-azaC in hydrogen peroxide-treated mouse insulinoma cells. HP stimulation served as a model of oxidative stress and stress-induced premature senescence (SIPS) (**left**). HP (H_2_O_2_) treatment resulted in increased levels of Trdmt1, ROS, RNA methylation (5-mC and m^6^A pools), cell cycle arrest at the G2/M phase and increased senescence-associated β-galactosidase (SA-β-gal) activity (**left**). 5-AzaC post-treatment prevented the development of senescence phenotype in both insulinoma cell lines. In HP-treated β-TC-6 cells, 5-azaC post-treatment promoted nitric oxide-mediated necrotic cell death and cytotoxic autophagy that was accompanied by increased levels of p-eIF2α and LC3B and diminished levels of RNA methylation parameters (**right**). The levels of Trdmt1 were elevated after 5-azaC post-treatment, but Trdmt1 cannot take part in an adaptive stress response as a result of the inhibitory action of 5-azaC. Thus, this approach can sensitize oxidant-stressed insulinoma cells to 5-azaC treatment resulting in cell death.

In conclusion, we have investigated for the first time the pleiotropic effects of 5-azaC against two different insulinoma cell lines under oxidative stress conditions (Figure 7).

5-AzaC-mediated modulation of senescence, apoptotic and autophagic responses were considered. 5-AzaC post-treatment prevented the development of the HP-induced senescence phenotype and did not potentiate HP-stimulated apoptosis in insulinoma cells. Anti-senescence action of 5-azaC may be important in limiting β-cell senescence-mediated implications in diabetes development. On the contrary, 5-azaC post-treatment induced autophagy was associated with nitric oxide-mediated necrosis in β-TC-6 cells (Figure 7). We suggest that 5-azaC-based cytotoxic autophagy in HP-stressed β-TC-6 cells may promote the elimination of insulinoma cells that may be considered a novel experimental approach in anticancer therapy, at least against insulinoma cells in vitro. More studies are also needed to document the action of 5-azaC in oxidizing conditions in in vivo models. As we used two insulinoma cell lines NIT-1 and β-TC-6 cells, which are derived from different mouse strains and considered as T1D and T2D cellular models, respectively, one can speculate that observed diverse responses to combined treatment of HP and 5-azaC can rely on different genetic backgrounds of two insulinoma cell lines.

## Data Availability

Data are available from the corresponding author on reasonable request.
